# Effectiveness of the 10 Essentials Workshop Program in Changing Attitudes Toward Suicide Among Mental Health Professionals

**DOI:** 10.7759/cureus.77830

**Published:** 2025-01-22

**Authors:** Kenji Narita, Chiaki Kawanishi, Yusuke Tsuyama, Shizuka Kawamoto, Tomonori Kashiwagi, Ryutaro Ishibashi, Yoshinori Cho, Kotaro Otsuka

**Affiliations:** 1 Neuropsychiatry, Sapporo Medical University Graduate School of Medicine, Sapporo, JPN; 2 General Education, Kyoto Seika University, Kyoto, JPN; 3 National Institute of Mental Health, National Center of Neurology and Psychiatry, Tokyo, JPN; 4 Neuropsychiatry, Iwate Medical University, Morioka, JPN

**Keywords:** attitude, education program, mental health professional, suicidal behavior, suicide prevention

## Abstract

Introduction: Mental health professionals play an important role in helping patients at risk of suicide. Therefore, mental health professionals must acquire basic knowledge and skills in suicide prevention to support patients with suicidal behavior. However, not all mental health professionals have sufficient knowledge and skills, and educational programs for mental health professionals are rare. We previously developed a workshop covering 10 steps healthcare professionals must take to care for mentally ill patients (the 10 Essentials program), and we adapted it for suicide prevention. This study aimed to evaluate the 10 Essentials program's effectiveness in educating mental health professionals.

Methods: This was an intervention study (pre-post comparison test). The participants of the present study were the mental health professionals (psychiatrists, psychiatric nurses, mental health social workers, clinical psychologists, and occupational therapists) who took part in the 10 Essentials workshops we delivered to several communities in Japan. The Attitudes Toward Suicide Scale (ATTS) was administered to the participants pre- and post-training to assess changes in their attitudes toward suicide after participating in the program.

Results: We analyzed data from 264 participants who completed the questionnaires. Their ATTS scores at post-training were significantly higher (and therefore more favorable) in the following dimensions: “common occurrence” (p = 0.012), “unjustified behavior” (p < 0.0001), and “preventability/readiness to help” (p < 0.0001), compared with those at pretraining. The ATTS scores at post-training were significantly lower (and therefore more favorable) in “right to suicide” (p < 0.0001), “suicidal expression as a mere threat” (p < 0.0001), and “impulsiveness” (p < 0.0001) compared with pretraining scores. Attitudes toward suicide among the participants shifted in a favorable direction after the program.

Conclusion: In this study, the 10 Essentials program was effective in improving attitudes toward suicide among mental health professionals. Their attitudes became more favorable for helping patients with suicidal behavior. Further study will be needed to examine participants with different backgrounds and the long-term effects of the training.

## Introduction

Training for mental health professionals

Suicide is a major public health issue worldwide. The World Health Organization (WHO) reported that an estimated 800,000 people die by suicide each year globally [1]. Several risk factors have been identified to date, and medical professionals commonly encounter patients who are at risk for suicide [1]. Mental health professionals play an especially important role, often providing the frontline response to suicidal patients because suicidal behaviors are affected by mental disorders [2,3]. To provide appropriate and effective intervention for suicidal individuals, mental health professionals require specialized knowledge. Specifically, they need to understand evidence-based suicide risk factors, including diagnostic, demographic, and psychosocial factors (life events, pressures and expectations, social isolation, sudden changes in social systems, and absence of protective factors), and they need the skills to manage these factors by a comprehensive approach, including emotional support, crisis intervention, and access to mental health services [4]. However, not all mental health professionals have sufficient knowledge and skills to provide appropriate care for individuals at risk for suicide [5]. It might be due to the lack of training for suicide prevention in the education competencies. Although there exist several suicide-related educational programs for mental health professionals, few comprehensively cover risk assessment, risk management, treatment, and service planning, and reports on the effectiveness of such programs are scarce in the literature [6]. We developed an educational program, called 10 Essentials, to train mental health professionals (psychiatrists, psychiatric nurses, mental health social workers, clinical psychologists, and occupational therapists) on necessary primary care for mental illness and suicide risk. The 10 Essentials program is unique in the breadth of its coverage, including issues such as communicating with and providing support to patients’ families and others (Appendix A). The program is conducted by mental health professionals, including psychiatrists, psychiatric nurses, mental health social workers, and clinical psychologists, and can be adapted for suicide prevention. The 10 Essentials program is summarized as the following: the 10 steps on a time sequence that capture the essence required to support suicidal individuals: notice suicidal people, talk warmly, listen with sympathy and trust, identify suicide risks, ensure their safety, solve problems, set up information, encourage them to contact support services, and act as a mediator, self-help, and assist the family. The program deals with a comprehensive approach that includes the support network, such as the family and the community, together with its formal, informal, and natural leaders. This study aimed to evaluate the 10 Essentials program's effectiveness in educating mental health professionals.

## Materials and methods

Participants

In Japan, a big surge in the number of suicides occurred in 1998 (32,863 victims compared to 24,391 in 1997), and it had stayed at a high level for years [2]. In response, The General Principles of Suicide Prevention in Japan was revised in 2017 by The Basic Act on Suicide Prevention [7], thereby every prefecture and municipality must plan and implement suicide prevention measures in their communities. Recruiting and training personnel engaged in suicide countermeasures is one of the priorities described in The General Principles of Suicide Prevention. Accordingly, several local governments asked us to develop and conduct educational workshops on suicide prevention for mental healthcare professionals, including psychiatrists, psychiatric nurses, mental health social workers, clinical psychologists, and occupational therapists. The participants of the present study are healthcare professionals who took part in one of these workshops in their respective communities (Nagoya, Kobe, Niigata, Kyoto, Fukuoka, Yokohama, and Sapporo) between July 1, 2017, and December 31, 2023. This study included all participants who consented to the study, with no specific exclusion criteria applied. Each participant provided written informed consent prior to enrollment.

Educational program

The 10 Essentials-based suicide prevention workshop consists of five parts: 1) a basic lecture on suicide and suicide behaviors, 2) an introduction to the 10 Essentials Program, 3) a case study, 4) a review lecture, and 5) a question-and-answer and discussion (Table 1).

**Table 1:** Agenda of suicide prevention workshop based on 10 essentials

	Content	Time (minutes)
1	Welcome	10
2	Basic lecture on suicide and suicide behaviors	60
3	Introduction of the 10 Essentials Program	30
4	Case study according to 10 Essentials Program	90
5	Review lecture	20
6	Lecture on postvention of suicide	20
7	Q&A and discussion	20
8	Closing remarks	10

These workshops were held in the meeting room, and the participants were divided into several clusters for case scenario sessions. The developers of the program (the psychiatrists) led the workshops. The basic lecture covers the epidemiology of suicide, suicide risk factors, psychological aspects of suicidal individuals, and an introduction to the concept of suicide prevention. This modified 10 Essentials program was developed by Otsuka and Kawanishi for frontline health professionals in a position to intervene with suicidal individuals. The program consists of 10 learning requirements, which were developed with reference to WHO publications [8,9] and Mental Health First Aid [10]. The program describes 10 essential steps in the process of caring for suicidal individuals. Participants receive a booklet introducing the concept of suicide prevention and describing the 10 steps. These are written in a suicidal-person-centered manner and focus on the knowledge and practical skills necessary for mental healthcare professionals to intervene (Appendix B). The case study is the longest section of the workshop. It is based on a fictional scenario designed to teach participants best practices for applying the steps in the 10 Essentials framework to intervene with suicidal individuals. The scenario deals with a special case about a patient who repeats self-harm and has multiple risk factors for suicide at the same time. This interactive exercise is conducted in small groups of 4-6 participants with a facilitator. The facilitator encourages the participants to consider how to manage the case and solve problems step by step.

Outcome measures

Participants completed the Attitudes Toward Suicide Scale (ATTS) at two time points: immediately before the workshop (pretraining) and immediately after the workshop (post-training). ATTS is a self-administered questionnaire developed in Sweden to measure attitudes toward suicide [11]. The ATTS can be utilized in large survey studies, and we found it to be the most feasible among all scales used to assess the same construct. The ATTS scale consists of 37 items scored on a five-point scale from 1 (disagree completely) to 5 (agree completely). It was developed to measure the following dimensions of attitudes toward suicide: “right to suicide,” “common occurrence,” “suicidal expression as mere threat,” “unjustified behavior,” “preventability/readiness to help,” and “impulsiveness.” The Japanese version of the ATTS was developed using the standard backtranslation model recommended by Brislin [12,13]. Participants answered questions regarding the following demographic and profession-related factors at pretraining: gender, age, current occupation, number of years working in current occupation, number of experiences caring for suicidal patients in the past year, and history of participation in suicide prevention training.

Statistical analysis

We summarized data by means, standard deviations, numbers, and proportions. We analyzed differences between pre- and post-training ATTS scores using the t-test. Multivariate regression analysis was used to examine factors associated with changes in ATTS scores between pre- and post-training. We calculated regression coefficients (β) and 95% confidence intervals (CIs). We used the statistical analysis software JMP Pro 17 (SAS Institute, Inc., Gary, USA).

Ethics approval

The present study was approved by the Ethical Committee of Sapporo Medical University (approval number: 3-1-23).

## Results

During the study period, 283 mental health professionals attended the suicide prevention workshops. After excluding those with missing data in their ATTS responses, 264 participants were included for analysis. The characteristics of the participants are shown in Table 2.

**Table 2:** Characteristics of participants at pretraining (n = 264)

	Numbers (%)
Gender	
Male	140 (53.0)
Female	124 (47.0)
Age	
20-29	36 (13.6)
30-39	73 (27.7)
40-49	78 (29.6)
50-59	49 (18.6)
60-69	22 (8.3)
70-79	6 (2.3)
Current occupation	
Psychiatrist	127 (48.1)
Psychiatric nurse	61 (23.1)
Mental health social worker	37 (14.0)
Other^a^	39 (14.8)
Number of years working as current occupation (in years)	
<1	8 (3.0)
01-May	58 (22.0)
06-Oct	53 (20.1)
>10	145 (54.9)
Experience with caring for suicidal patients in the past year	
0-10 patients	163 (61.7)
≧11 patients	101 (38.3)
History of participation in suicide prevention training	
With experience	140 (53.0)
Without experience	124 (47.0)
^a ^Clinical psychologist or occupational therapist	

For anonymization purposes, individuals were not asked their exact age but their age range. Therefore, the age variable is distributed by age group. The results of the comparison of pre- and post-training ATTS scores are shown in Table 3.

**Table 3:** Differences in ATTS scores between pre- and post-training (n = 264) ATTS: Attitudes Toward Suicide Scale

Category	Pretraining mean (SD)	Post-training mean (SD)	Mean difference (SD)	p-value*
Right to suicide	2.58 (0.66)	2.23 (0.65)	-0.35 (0.53)	<0.0001
Common occurrence	3.42 (0.56)	3.50 (0.57)	0.08 (0.49)	0.012
Suicidal expression as mere threat	1.93 (0.77)	1.48 (0.63)	-0.46 (0.71)	<0.0001
Unjustified behavior	3.27 (0.90)	3.49 (0.98)	0.22 (0.84)	<0.0001
Preventability/readiness to help	3.54 (0.62)	4.00 (0.63)	0.46 (0.60)	<0.0001
Impulsiveness	2.81 (0.53)	2.65 (0.62)	-0.16 (0.59)	<0.0001

Participants’ ATTS scores at post-training were significantly higher in the dimensions of the “common occurrence” (p = 0.012), “unjustified behavior” (p < 0.0001), and “preventability/readiness to help” (p < 0.0001) than those at pretraining. The ATTS scores at post-training were significantly lower in the dimensions of “right to suicide” (p < 0.0001), “suicidal expression as mere threat” (p < 0.0001), and “impulsiveness” (p < 0.0001) than those at pretraining. The pre- and post-training ATTS scores for each characteristic are shown in Table 4.

**Table 4:** ATTS scores at pre- and post- training ATTS: Attitudes Toward Suicide Scale

		Right to suicide		Common occurrence		Suicidal expression as mere threat		Unjustified behavior		Preventability/readiness to help		Impulsiveness	
		Pre (mean (SD))	Post (mean (SD))	Pre (mean (SD))	Post (mean (SD))	Pre (mean (SD))	Post (mean (SD))	Pre (mean (SD))	Post (mean (SD))	Pre (mean (SD))	Post (mean (SD))	Pre (mean (SD))	Post (mean (SD))
Gender													
Male		2.63 (0.68)	2.28 (0.64)	3.48 (0.54)	3.54 (0.56)	1.84 (0.76)	1.45 (0.61)	3.32 (0.95)	3.52 (1)	3.55 (0.64)	3.96 (0.68)	2.81 (0.53)	2.68 (0.6)
Female		2.52 (0.64)	2.18 (0.65)	3.36 (0.58)	3.45 (0.58)	2.04 (0.77)	1.51 (0.64)	3.22 (0.84)	3.46 (0.97)	3.53 (0.6)	4.05 (0.56)	2.81 (0.53)	2.63 (0.64)
Age													
20-29		2.77 (0.56)	2.33 (0.61)	3.54 (0.62)	3.71 (0.41)	2.17 (0.83)	1.57 (0.62)	3.06 (0.72)	3.35 (0.78)	3.62 (0.47)	4.07 (0.57)	2.73 (0.58)	2.54 (0.62)
30-39		2.65 (0.66)	2.24 (0.6)	3.47 (0.51)	3.5 (0.55)	1.91 (0.71)	1.44 (0.59)	3.2 (0.95)	3.29 (1.01)	3.5 (0.59)	4 (0.64)	2.74 (0.55)	2.61 (0.57)
40-49		2.57 (0.67)	2.24 (0.72)	3.43 (0.52)	3.55 (0.53)	1.89 (0.8)	1.46 (0.62)	3.17 (0.91)	3.53 (0.98)	3.47 (0.68)	3.96 (0.66)	2.76 (0.53)	2.62 (0.64)
50-59		2.4 (0.69)	2.12 (0.68)	3.36 (0.58)	3.38 (0.65)	1.87 (0.78)	1.41 (0.69)	3.7 (0.74)	3.79 (0.94)	3.66 (0.64)	4.13 (0.6)	2.96 (0.4)	2.74 (0.68)
60-69		2.46 (0.68)	2.22 (0.56)	3.26 (0.71)	3.3 (0.77)	1.91 (0.78)	1.66 (0.66)	3.36 (0.97)	3.68 (0.93)	3.53 (0.67)	3.82 (0.6)	3 (0.51)	2.91 (0.5)
70-79		2.56 (0.6)	2.17 (0.71)	3.13 (0.47)	3.2 (0.36)	1.92 (0.74)	1.5 (0.63)	2.92 (1.2)	3.08 (1.72)	3.61 (0.68)	3.83 (0.66)	2.94 (0.83)	2.78 (0.5)
Current occupation													
Psychiatrist		2.62 (0.76)	2.24 (0.7)	3.4 (0.56)	3.44 (0.57)	1.8 (0.74)	1.41 (0.57)	3.33 (0.92)	3.53 (1.01)	3.56 (0.64)	3.99 (0.65)	2.89 (0.49)	2.71 (0.59)
Psychiatric nurse		2.59 (0.52)	2.25 (0.59)	3.39 (0.61)	3.47 (0.59)	2.21 (0.81)	1.74 (0.75)	3.34 (0.8)	3.54 (0.85)	3.41 (0.61)	3.98 (0.67)	2.88 (0.54)	2.71 (0.68)
Mental health social worker		2.51 (0.64)	2.21 (0.66)	3.52 (0.53)	3.62 (0.55)	2.15 (0.8)	1.49 (0.61)	3.1 (0.86)	3.34 (0.94)	3.64 (0.51)	4.05 (0.55)	2.62 (0.53)	2.57 (0.58)
Others		2.49 (0.52)	2.18 (0.57)	3.44 (0.51)	3.6 (0.56)	1.72 (0.63)	1.28 (0.52)	3.15 (1.01)	3.42 (1.13)	3.61 (0.64)	4.04 (0.57)	2.62 (0.56)	2.46 (0.61)
Experience with caring for suicidal patients in the past year													
0-10 patients		2.53 (0.66)	2.21 (0.65)	3.4 (0.57)	3.52 (0.59)	2.01 (0.8)	1.48 (0.64)	3.35 (0.88)	3.49 (1)	3.52 (0.61)	4.01 (0.62)	2.78 (0.57)	2.62 (0.63)
≧11 patients		2.65 (0.66)	2.26 (0.65)	3.46 (0.55)	3.46 (0.55)	1.8 (0.71)	1.47 (0.61)	3.14 (0.93)	3.48 (0.96)	3.58 (0.64)	3.99 (0.65)	2.86 (0.47)	2.7 (0.6)
History of participation in suicide prevention training													
With experience		2.58 (0.61)	2.25 (0.59)	3.46 (0.55)	3.56 (0.56)	1.84 (0.75)	1.46 (0.6)	3.19 (0.92)	3.44 (0.98)	3.63 (0.64)	4.07 (0.61)	2.8 (0.53)	2.66 (0.59)
Without experience		2.58 (0.72)	2.21 (0.72)	3.37 (0.57)	3.42 (0.58)	2.04 (0.78)	1.5 (0.65)	3.36 (0.87)	3.54 (0.98)	3.45 (0.58)	3.93 (0.63)	2.83 (0.54)	2.65 (0.64)

Table 5 shows the results of a multivariate regression analysis based on the difference in the pre-post scores of ATTS for each characteristic.

**Table 5:** Multivariate regression analysis of differences in ATTS scores ATTS: Attitudes Toward Suicide Scale

		Right to suicide				Common occurrence				Suicidal expression as mere threat			
		Beta	95% CI		p-value	Beta	95% CI		p-value	Beta	95%CI		p-value
		Gender	Male	Reference				Reference				Reference			
	Female	-0.03	-0.17	0.11	0.663	0.00	-0.13	0.13	0.982	-0.09	-0.28	0.10	0.35
Age													
	20-29	Reference				Reference				Reference			
	30-39	0.08	-0.14	0.31	0.467	-0.10	-0.31	0.11	0.328	-0.02	-0.33	0.28	0.888
	40-49	0.17	-0.08	0.43	0.178	-0.01	-0.24	0.23	0.950	0.01	-0.34	0.35	0.972
	50-59	0.24	-0.09	0.56	0.149	-0.10	-0.40	0.20	0.509	-0.09	-0.52	0.34	0.685
	60-69	0.33	-0.12	0.78	0.145	-0.03	-0.44	0.39	0.904	0.01	-0.59	0.62	0.971
	70-79	0.21	-0.44	0.86	0.528	0.06	-0.54	0.65	0.856	-0.30	-1.18	0.57	0.496
Current occupation													
	Psychiatrist	Reference				Reference				Reference			
	Psychiatric nurse	0.08	-0.10	0.25	0.391	0.00	-0.15	0.16	0.951	-0.02	-0.25	0.22	0.884
	Mental health social worker	0.11	-0.11	0.33	0.319	-0.04	-0.24	0.17	0.712	-0.23	-0.53	0.06	0.122
	Others	0.09	-0.12	0.30	0.390	0.05	-0.14	0.24	0.572	0.01	-0.27	0.29	0.953
Number of years working as current occupation	Per one year increased	0.00	-0.01	0.01	0.766	0.00	-0.01	0.01	0.673	0.01	-0.01	0.02	0.366
Experience with caring for suicidal patients in the past year													
	0-10 patients	Reference				Reference				Reference			
	≧11 patients	-0.08	-0.22	0.07	0.303	-0.13	-0.27	0.00	0.048	0.11	-0.09	0.30	0.287
History of participation in suicide prevention training													
	Without experience	Reference				Reference				Reference			
	With experience	0.03	-0.11	0.17	0.650	0.09	-0.04	0.22	0.169	0.17	-0.02	0.37	0.072

		Unjustified behavior				Preventability/readiness to help				Impulsiveness			
		Beta	95% CI		p-value	Beta	95% CI		p-value	Beta	95% CI		p-value
		Gender	Male	Reference				Reference				Reference			
	Female	0.04	-0.18	0.26	0.739	0.11	-0.05	0.27	0.162	-0.06	-0.22	0.10	0.460
Age													
	20-29	Reference				Reference				Reference			
	30-39	-0.30	-0.66	0.06	0.099	0.11	-0.15	0.36	0.421	0.08	-0.18	0.33	0.566
	40-49	-0.09	-0.49	0.31	0.664	0.12	-0.16	0.41	0.401	0.06	-0.22	0.35	0.662
	50-59	-0.46	-0.97	0.05	0.078	0.13	-0.23	0.50	0.476	-0.03	-0.40	0.33	0.866
	60-69	-0.37	-1.08	0.34	0.306	0.00	-0.50	0.51	0.993	0.12	-0.39	0.63	0.644
	70-79	-0.72	-1.75	0.31	0.168	0.03	-0.71	0.76	0.943	0.01	-0.73	0.75	0.983
Current occupation													
	Psychiatrist	Reference				Reference				Reference			
	Psychiatric nurse	0.04	-0.24	0.31	0.782	0.07	-0.13	0.27	0.484	0.04	-0.16	0.24	0.683
	Mental health social worker	0.07	-0.28	0.42	0.704	-0.12	-0.37	0.13	0.336	0.18	-0.07	0.43	0.165
	Others	0.10	-0.22	0.43	0.542	-0.09	-0.33	0.14	0.430	0.05	-0.18	0.29	0.649
Number of years working as current occupation	Per one year increased	0.01	-0.01	0.03	0.172	-0.01	-0.02	0.01	0.361	0.00	-0.01	0.01	0.793
Experience with caring for suicidal patients in the past year													
	0-10 patients	Reference				Reference				Reference			
	≧11 patients	0.20	-0.03	0.43	0.081	-0.07	-0.23	0.10	0.422	-0.01	-0.17	0.16	0.944
History of participation in suicide prevention training													
	Without experience	Reference				Reference				Reference			
	With experience	0.03	-0.20	0.25	0.817	0.02	-0.14	0.18	0.801	0.01	-0.15	0.17	0.938

The analysis revealed a negative correlation between “common occurrence” scores and having 11 or more experiences with caring for suicidal patients in the past year when compared with those with 10 or fewer experiences (β = -0.13; 95% CI: -0.27-0.00; p = 0.048).

## Discussion

We examined the educational effectiveness of the 10 Essentials program among mental health professionals. In the present study, we assessed the six subscales constituting the ATTS. All participants already had preferable attitudes toward each subscale, and these attitudes significantly changed after the training. Because all participants were mental healthcare providers who engaged in the treatment or management of suicidal patients, most of them had the perception that most suicide attempters have mental disorders and that their suicide-related behaviors are caused by associated brain dysfunction [14]. Owing to their backgrounds, the participants tended to take the attitude of denying the “right to suicide” at pretraining. For the same reason, they were likely to agree with questions concerning “unjustified behavior” and “preventability/readiness to help” at pretraining. Although such trends had already been observed at pretraining, they were strengthened after the training. We conclude that the 10 Essentials program was effective, even for those who were regularly engaged in mental health care. Suicide is not an individual problem, but a social problem; anyone can become inclined to suicide [1]. Concerning suicide as a “common occurrence,” the participants of this study tended to believe that anyone could commit suicide. This tendency became stronger after the training. Although some might believe that those who often talk about their suicidal thoughts rarely commit suicide, this is a myth [15]. Expression of suicidal thoughts is a risk factor for suicide. The participants of this study were likely to take the attitude against the “suicidal expression as mere threat” at pretraining. This attitude was more apparent after the training. In fact, suicide does not occur suddenly, but follows a process. Most suicide attempters have mental disorders and related psychosocial problems [16], and their initial suicidal ideation might develop along a continuum of suicide plans, attempts, and death [17]. Therefore, mental health professionals need to understand that suicide attempts are not impulsive or inexplicable acts. Although the participants in this study may have already had a certain level of knowledge at pretraining, the 10 Essentials program enhanced the attitude that suicide is not an impulsive act. The multivariate regression analysis showed that those with 10 or fewer experiences caring for suicidal patients in the past year showed greater improvement in “common occurrence” scores than those with 11 or more experiences.

As shown in Table 4, it can be assumed that the group who had more experience with suicidal patients had higher baseline scores in “common occurrence” and was thus less likely to achieve change, whereas the group with less experience was more likely to show training effects. In the present study, except for the “common occurrence” dimension, no significant difference was seen in the scores of participants with different demographic characteristics. We conclude that the training was effective for all participants. As an educational program of suicide prevention for mental health professionals, only Skills-based Training on Risk Management (STORM) skill training [18] and Screening Tool for Assessing Risk of Suicide (STARS) protocol training [19] had multiple components and were reported to be effective. However, those programs targeted specific efficacies or competencies for suicide risk assessment. The unique feature of our training program is its coverage of support and management, including the action plan. In one study [20], it was reported that the participants’ attitudes toward suicide prevention, assessed by the Attitudes to Suicidal Prevention Scale, were preferable after training. However, attitudes toward suicide prevention were not divided into subscales in that study. In terms of workshops in Japan, the training programs on suicide prevention are limited [21,22], and, they are focused on earlier intervention, while this training session is more longitudinal and comprehensive. Additionally, while this program was developed in Japan, the contents of the program deal with common risk factors and universal knowledge related to suicide and could potentially be implemented outside of Japan. Further study will be required to investigate the feasibility of implementing this program internationally. This study had several limitations. First, participants represented several different types of occupation, and some occupational groups had small sample sizes for further analysis. Second, the sample was partly lost due to missing data in the questionnaire. While more than 93% of the respondents were included, the results did not reflect all who participated in the training. Third, we used only one scale for assessing the effectiveness of the education program. We considered it difficult to use more than one measure, taking into account the burden on participants. Fourth, information on the type of institution to which the participants belonged was not able to be collected. Finally, whereas the changes in attitude were assessed immediately after the training, the long-term effect of training was not assessed.

## Conclusions

We developed an educational program on necessary steps in primary care for mentally ill patients (the 10 Essentials program) and adapted it to focus on suicide prevention. The present study showed that the 10 Essentials program was effective for mental health professionals; it resulted in a change in their attitudes, which was preferable for helping patients with suicidal behavior. Additionally, no significant difference was seen in the improvement of the scores in “unjustified behavior,” “preventability/readiness to help,” “right to suicide,” “suicidal expression as mere threat,” and “impulsiveness” with different demographic characteristics. The program was effective for all participants regardless of their characteristics. Further study will be needed to examine participants with different professional backgrounds and to assess the long-term effects of training. International implementation of this program is also a future prospect, as the content deals with common risk factors and universal knowledge related to suicide and has the potential to be implemented outside of Japan.

## Figures and Tables

**Figure 1:**
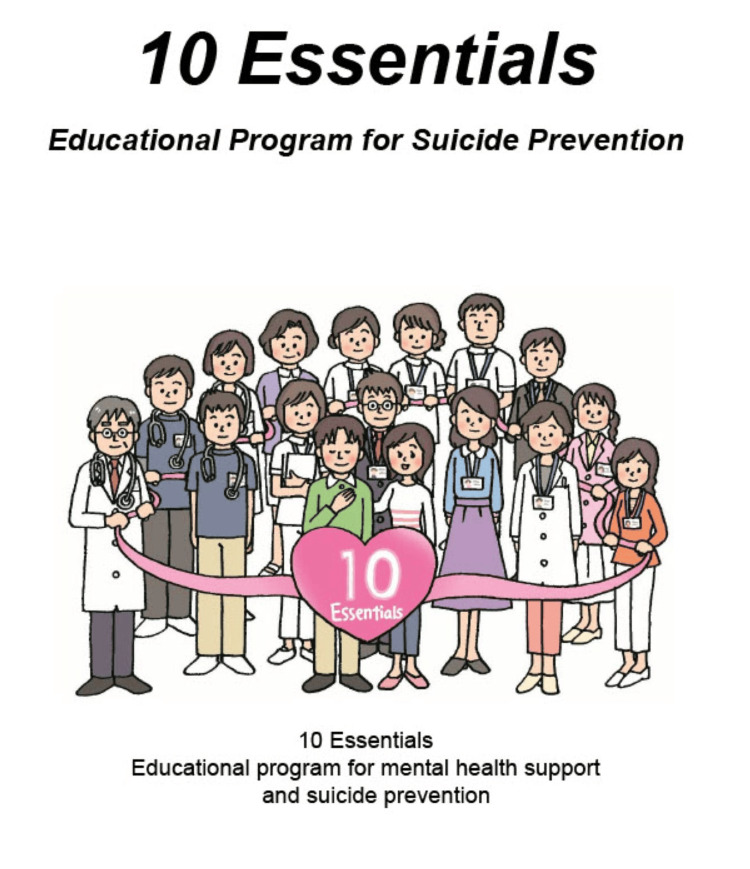
The 10 Essentials program: page 1

**Figure 2:**
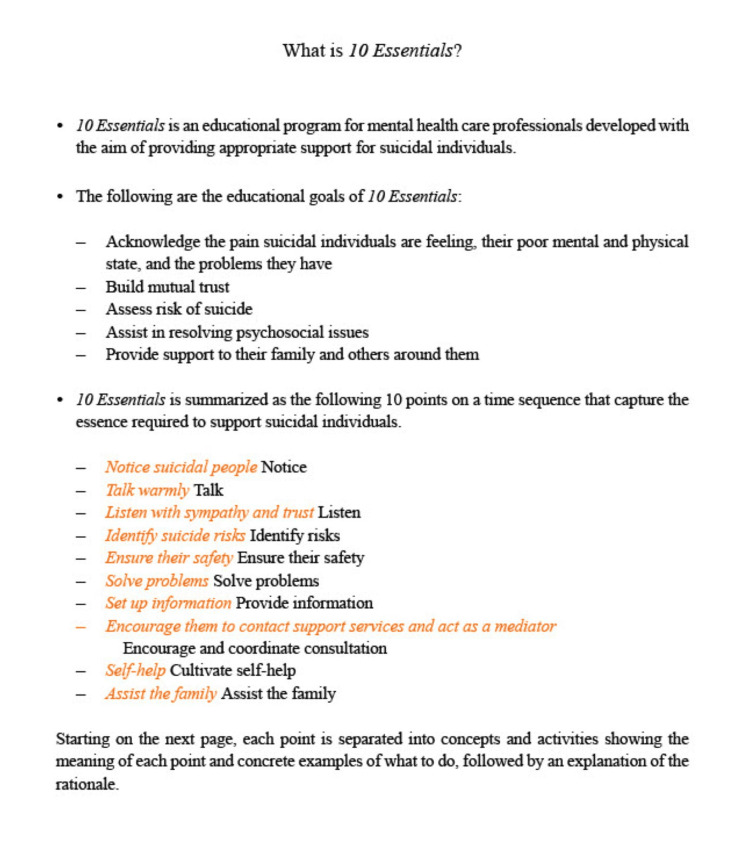
The 10 Essentials program: page 2

**Figure 3:**
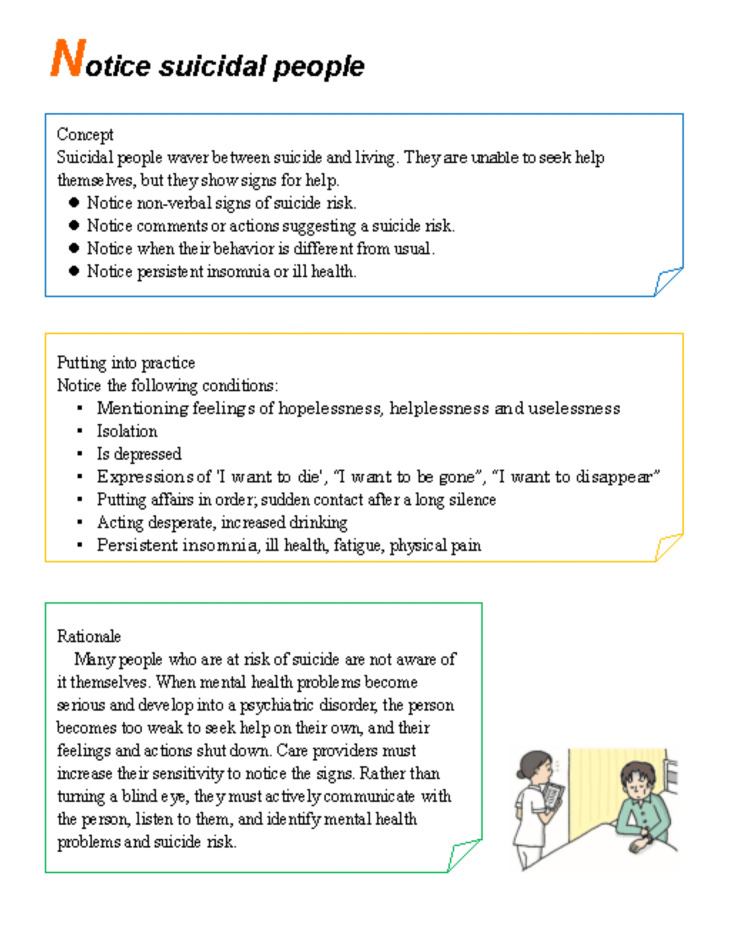
The 10 Essentials program: page 3

**Figure 4:**
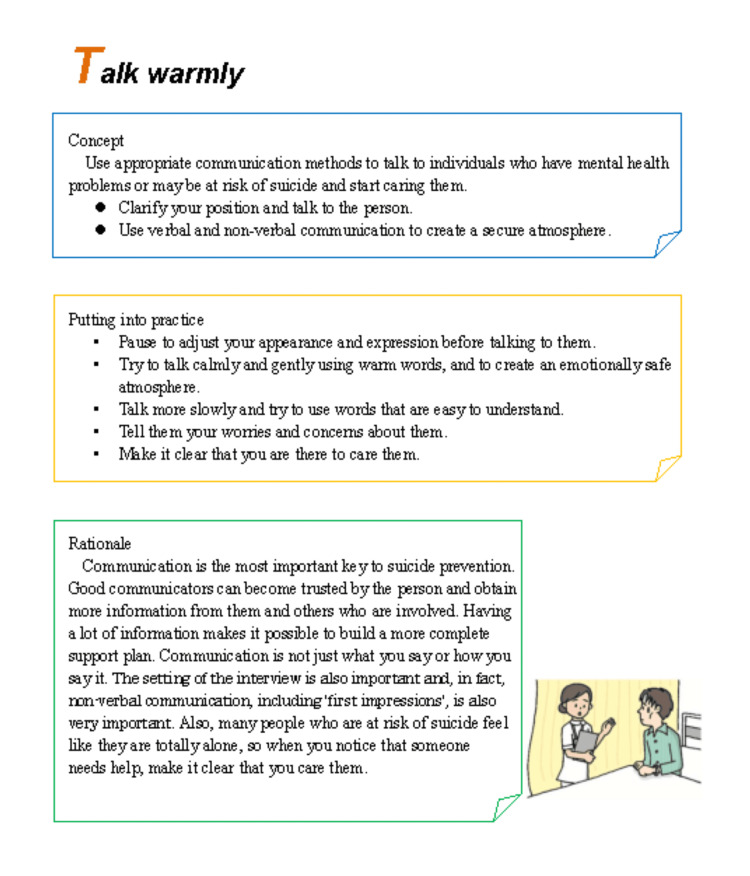
The 10 Essentials program: page 4

**Figure 5:**
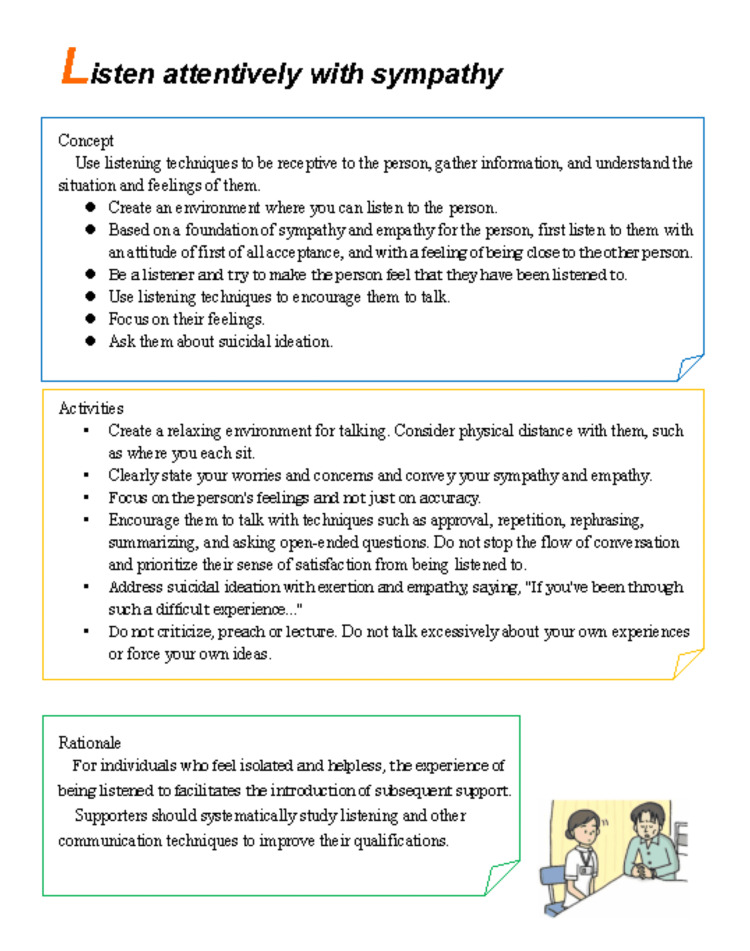
The 10 Essentials program: page 5

**Figure 6:**
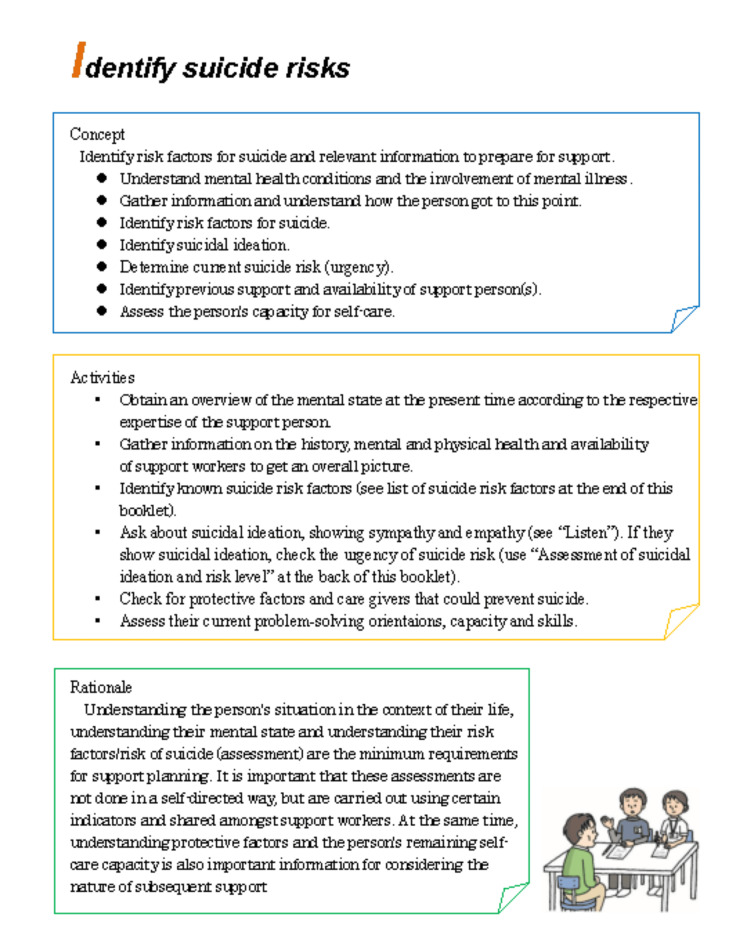
The 10 Essentials program: page 6

**Figure 7:**
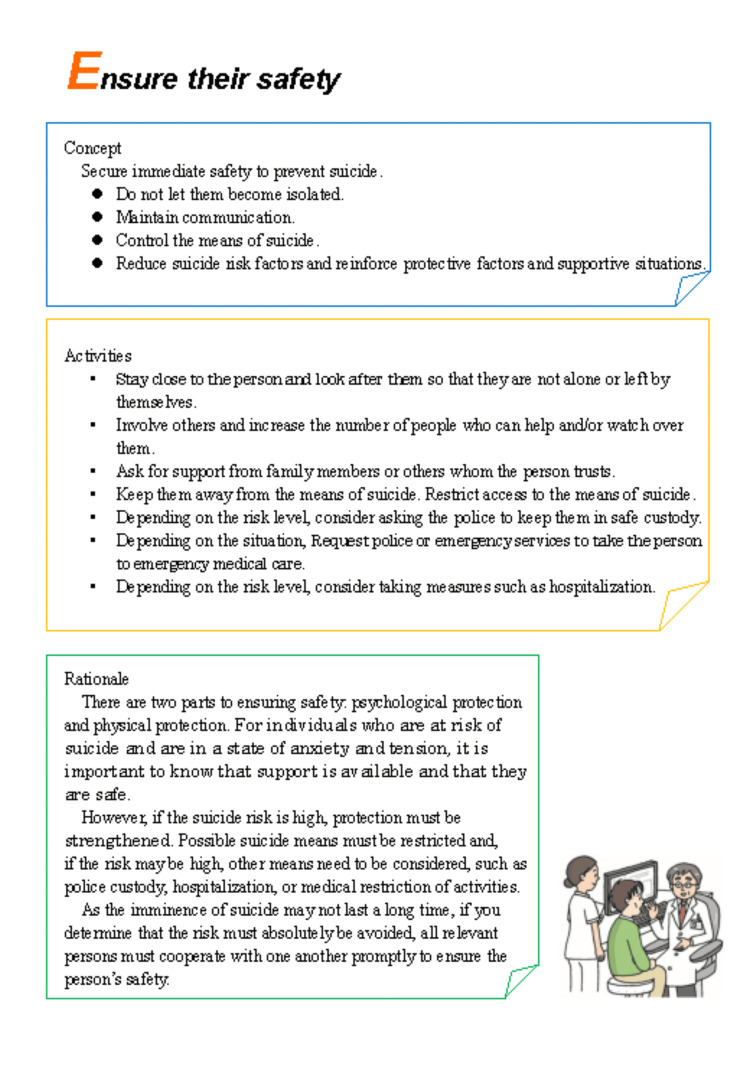
The 10 Essentials program: page 7

**Figure 8:**
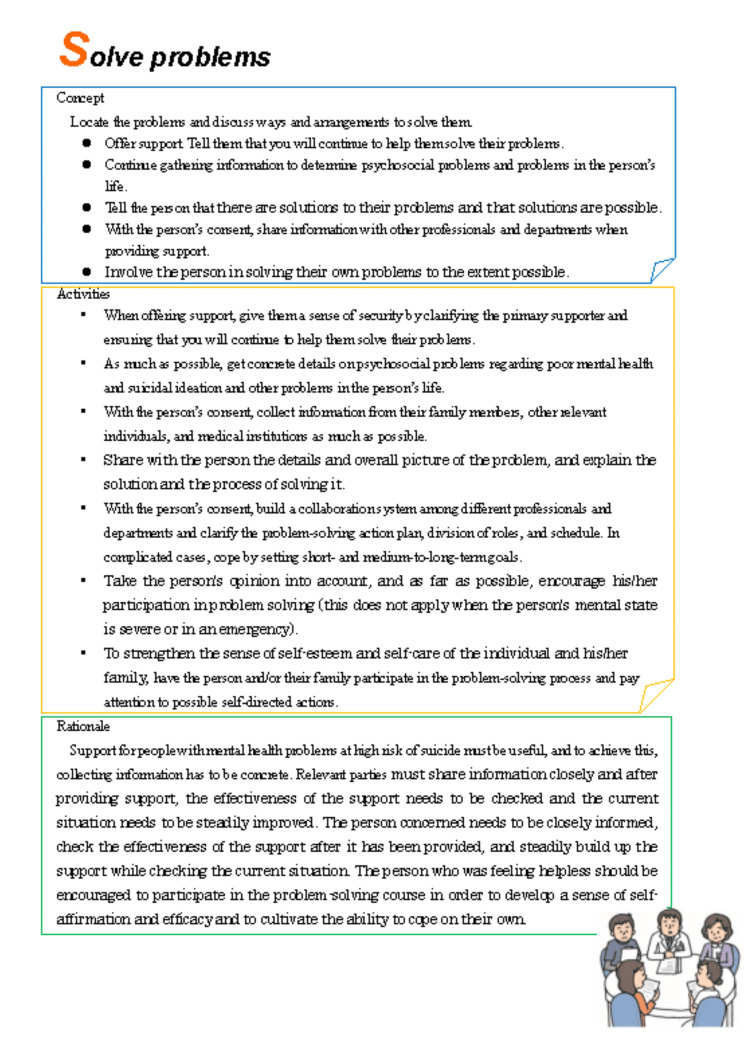
The 10 Essentials program: page 8

**Figure 9:**
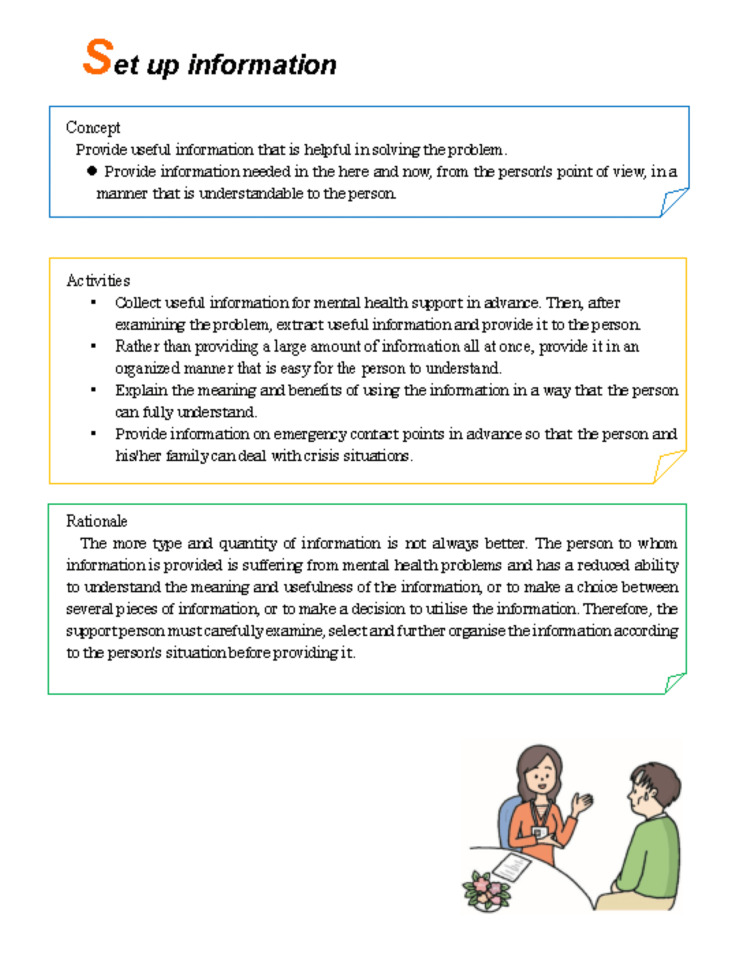
The 10 Essentials program: page 9

**Figure 10:**
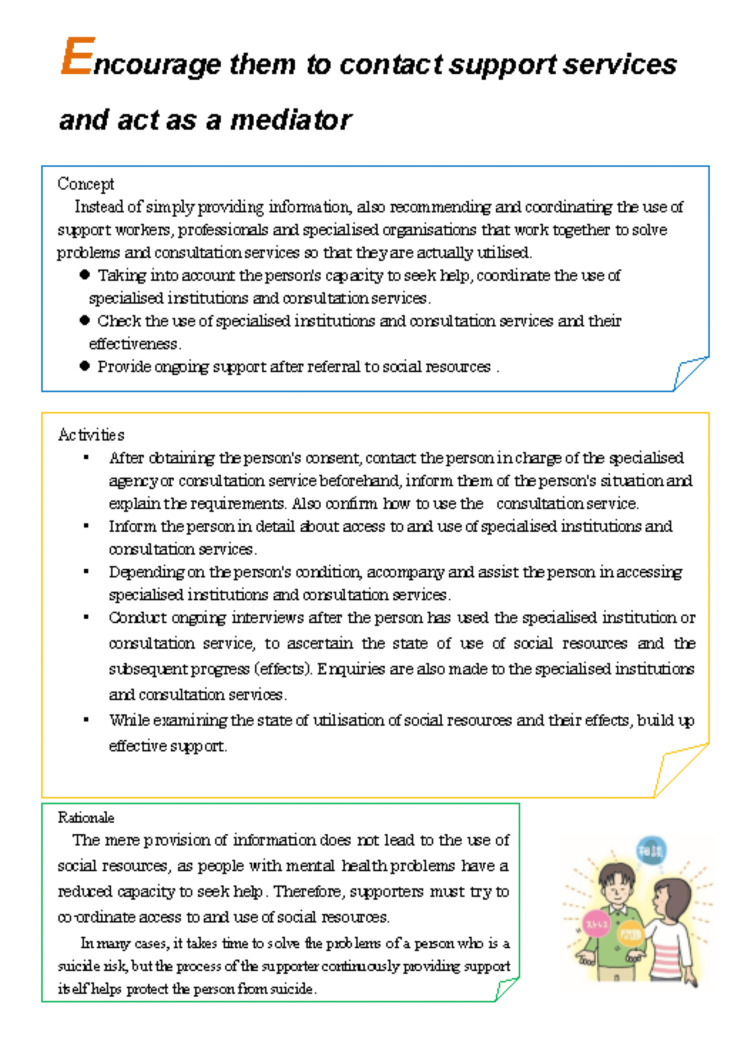
The 10 Essentials program: page 10

**Figure 11:**
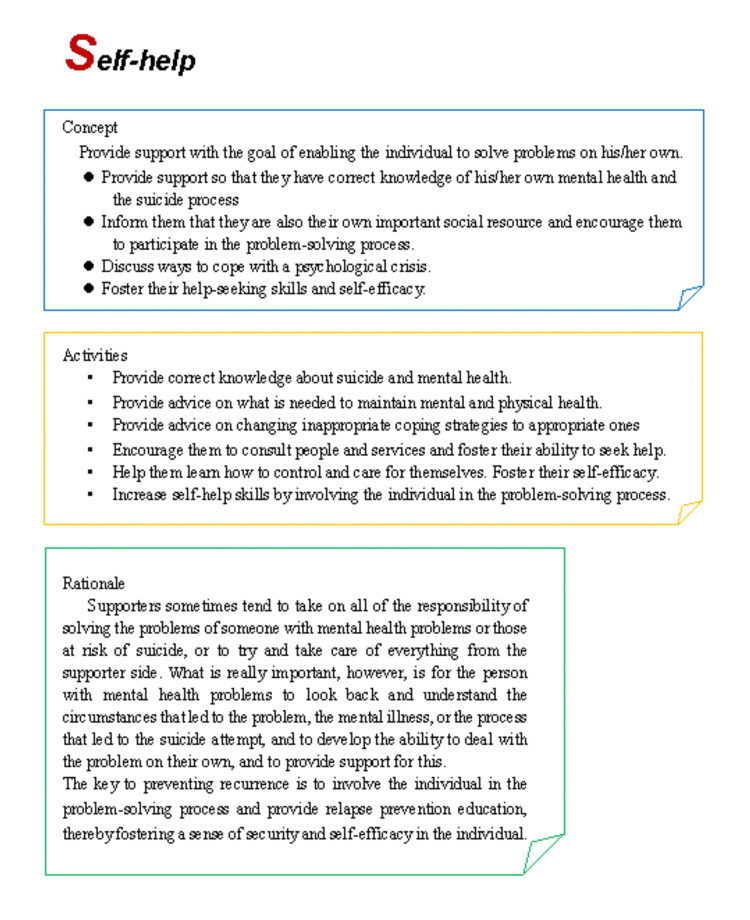
The 10 Essentials program: page 11

**Figure 12:**
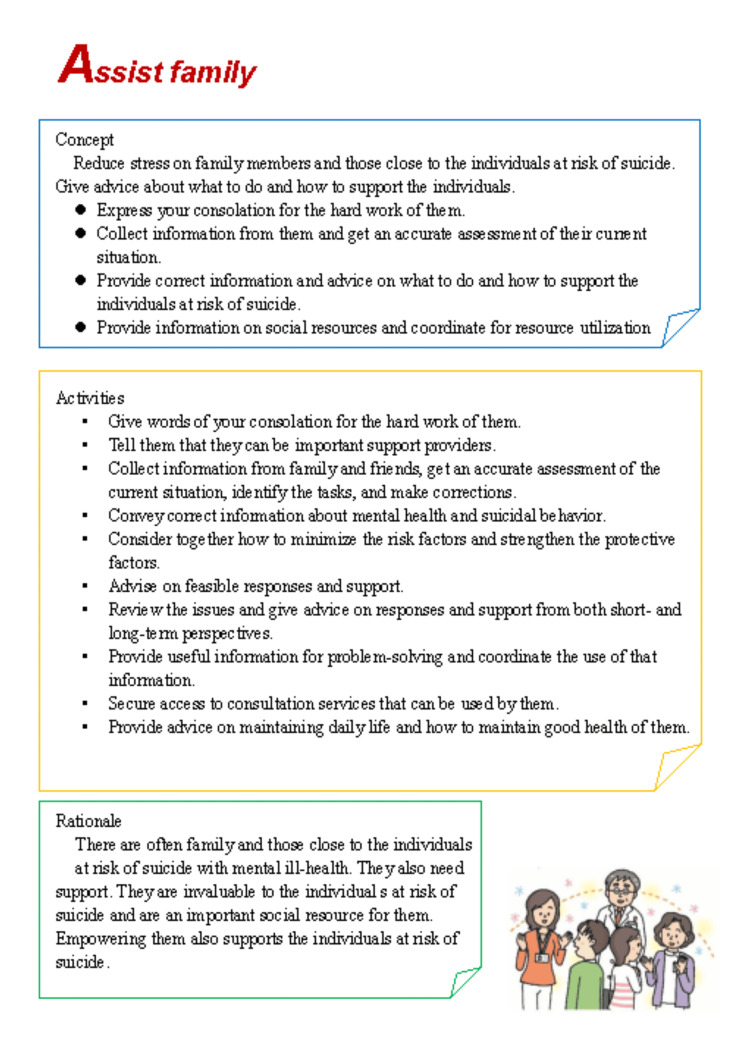
The 10 Essentials program: page 12

## Appendices

Appendix A 

Appendix B 

**Table 6:** The Japanese version of Attitudes Toward Suicide Scale (backtranslation)

		Storongly Agree	Agree	Undecided	Disagree	Strongly Disagree
1	Can always help those who are suicidal					
2	Suicide is never justified					
3	Suicide is the worst thing to do to relatives					
4	Suicide attempts are impulsive					
5	Suicide is an acceptable way to end incurable disease					
6	Suicide is carried out after long-term consideration					
7	People who make suicide threats rarely complete suicide					
8	Could kill myself out of loneliness					
9	Most people have thought about suicide					
10	Situations in which the only solution is suicide					
11	Could talk about my suicide wish without meaning it					
12	Suicide occurs without prior warning					
13	Do not understand others' wishes to commit suicide					
14	Should get help to die, if people suffer from severe, incurable disease					
15	I am ready to help suicidal people					
16	Anyone can commit suicide					
17	Can understand why people suffering from severe, incurable disease take their own lives					
18	People who talk about suicide do not actually take their lives					
19	Have a right to commit suicide					
20	Want to get help to die if I suffer from severe, incurable disease					
21	Suicide is preventable					
